# Enteral and Parenteral Nutrition in the Perioperative Period: State of the Art

**DOI:** 10.3390/nu5020608

**Published:** 2013-02-21

**Authors:** Salim Abunnaja, Andrea Cuviello, Juan A. Sanchez

**Affiliations:** Saint Mary’s Hospital, 56 Franklin Street, Waterbury, CT 06706, USA; E-Mails: ajcuviello@gmail.com (A.C.); juan.sanchez@stmh.org (J.A.S.)

**Keywords:** total parenteral nutrition, enteral nutrition, perioperative, immunonutrition

## Abstract

Nutritional support of surgical and critically ill patients has undergone significant advances since 1936 when Studley demonstrated a direct relationship between pre-operative weight loss and operative mortality. The advent of total parenteral nutrition followed by the extraordinary progress in parenteral and enteral feedings, in addition to the increased knowledge of cellular biology and biochemistry, have allowed clinicians to treat malnutrition and improve surgical patient’s outcomes. We reviewed the literature for the current status of perioperative nutrition comparing parenteral nutrition with enteral nutrition. In a surgical patient with established malnutrition, nutritional support should begin at least 7–10 days prior to surgery. Those patients in whom eating is not anticipated beyond the first five days following surgery should receive the benefits of early enteral or parenteral feeding depending on whether the gut can be used. Compared to parenteral nutrition, enteral nutrition is associated with fewer complications, a decrease in the length of hospital stay, and a favorable cost-benefit analysis. In addition, many patients may benefit from newer enteral formulations such as Immunonutrition as well as disease-specific formulations.

## 1. Introduction

Nutritional support of surgical and critically ill patients has undergone significant advances since 1936 when Studley demonstrated a direct relationship between preoperative weight loss and operative mortality [1,2]. Today, malnutrition is considered a risk factor for impaired systemic and intestinal immune function, as well as decreased digestive and absorptive capacity due to the altered architecture of the gut barrier [3]. The advent of total parenteral nutrition (TPN) followed by the extraordinary progress in parenteral and enteral feedings, in addition to the increased knowledge of cellular biology and biochemistry, have allowed clinicians to treat malnutrition and improve surgical patient’s outcomes [4]. This review will focus on the advantages, limitations, and comparisons of both parenteral and enteral nutrition in the malnourished perioperative patient.

Major stress, such as surgery, can subject a patient to a whole host of metabolic and physiologic changes. The body responds to such stress by increasing its basal metabolic rate (BMR), using up its nitrogen stores and creating a negative nitrogen balance [5]. An increase in gluconeogenesis as well as the synthesis of acute phase proteins is also observed [6]. The body scavenges for the required nutrients during such times of stress, which if continue unchecked for prolonged periods of time could lead to adverse consequences. Perioperative nutritional supplementation, therefore, should blunt the catabolic effects of such a high energy state [1]. Of interest is the increase in intestinal permeability during periods of surgical stress, which can be as such as fourfold greater in some patients usually normalizing around postoperative day five [1,7,8]. Associated with this increase in permeability, is a decrease in villous height, leading to malabsorption and an impaired ability of the gut to act as a barrier against endogenous bacteria and toxins [1,9]. Malnutrition and surgery can also both present a stress on the heart. Patients undergoing cardiac surgery are frequently found to be malnourished, resulting in alteration in the structure of myocytes and depleting the substrates utilized by the heart for mechanical work [10]. It is therefore hypothesized that by addressing the undernourished state of the patient prior to surgical intervention, we can improve cardiovascular performance function and minimize cardiac complications after surgery as well as lower perioperative mortality.

The most common surgical practice of making patients NPO (nil per os) after midnight of the day of any planned surgical procedure has been recently questioned. However; Brady *et al*. [11] reviewed 38 randomized controlled trials on perioperative fasting and concluded that there was no evidence to suggest overnight fasting for fluids results in a decrease in perioperative aspiration risk or related morbidities [11]. Evidence is emerging that overnight fasting is not just unnecessary, but may also be harmful. Surgical stress cause postoperative insulin resistance, immunosuppression, and increased patient discomfort [12,13]. Preoperative “carbohydrate loading” with carbohydrate rich drink three hours prior to scheduled procedure has been shown to attenuate the above adverse effects of fasting, particularly in diabetic patients [14,15,16,17].

Another previously prevalent practice involves the use of clear fluids only prior to surgery and in the early post operative period [6]. This can induce a starvation state as glycogen stores will be depleted within a few hours and the body promotes gluceoneogenesis through breakdown of muscle and other visceral proteins [6,10]. Perioperative nutritional support was, therefore, devised with the following goals: (1) to minimize negative protein balance by avoiding starvation, (2) to maintain muscle, immune, and cognitive function and, ultimately, (3) to enhance postoperative recovery and return of function [3]. Below we reviewed the literature for the current status of perioperative nutrition focusing on identifying the population at risk for malnutrition, comparing different forms of perioperative nutrition.

## 2. Nutritional Assessment and Population at Risk for Perioperative Malnutrition

Nutritional support is critical at a time of severe stress as the synthesis of acute phase proteins, white cells, fibroblasts, collagen, and other tissue components are required for proper wound healing and recovery [3,18,19,20]. In some circumstances energy requirements can reach as high as 30 kcal/kg ideal body weight, with a daily nitrogen requirement equivalent to a protein intake of 1.5 g/kg ideal body weight [3]. Preferably the protein:fat:glucose caloric ratio should approximate 20%:30%:50% of one’s daily intake [20]. It is therefore important for physicians to be able to determine which patients are at greater risk for postoperative complications and how malnourished these particular patients are prior to surgery.

**Figure 1:**
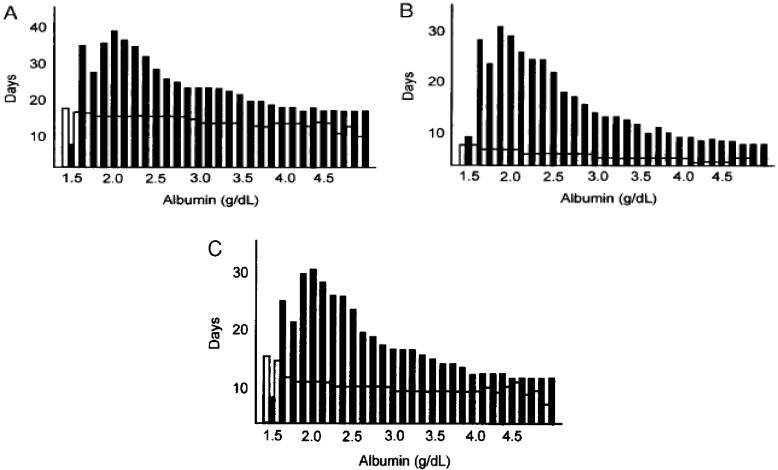
Postoperative stay (**A**), intensive care unit (ICU) stay (**B**), and nothing by mouth (NPO) days (**C**) remained relatively stable in the patients who recovered without complications, despite albumin level, except in the most hypoalbuminemic patients (open bars: patients with no complications; shaded bars: patients with complications). Note: This figure is reproduced with permission from [29], Copyright © 2003 The American Society for Parenteral and Enteral Nutrition.

Malnourishment is commonly seen in patients with an underlying illness such as cancer, or chronic organ failure [3,21,22,23,24,25,26,27,28]. For many years Albumin was of great interest for surgeons as an indicator of malnutrition. Figure 1 demonstrates a comparison between the serum albumin, an indicator of malnourishment, and the length of hospital stay for postoperative ICU and NPO patients, suggesting that malnutrition impact postsurgical outcomes. In this study, patients with an albumin of 3.25 g/dL the postoperative stay, ICU stay, and NPO days increased slightly with complications. As patients became more hypoalbuminemic, differences between patients with and without complications increased dramatically. The exception was the lowest albumin group who had short stays because of death after their complications [29].

However; neither serum nor urine proteins are specific nor sensitive indicators of malnutrition; since they can be highly influenced by other factors such as catabolism and fluid state [30,31]. In addition to serum albumin, surgeons and clinicians these days rely on several other clinical markers to identify those at risk for nutrition-related complications include substantial weight loss >10%–15% within 6 months, a very low BMI (<18.5–22 kg/m^2^), or evidence of acute inflammation [1,3,32,33,34]. The European Society for Clinical Nutrition and Metabolism (ESPEN) guidelines recommend the use of the Nutrition Risk Screening (NRS) 2002 tool, along with subjective global assessment, and serum albumin <30 g/L in their evaluation of undernutrition [30,35]. Table 1 illustrates the components of the tool. In one study by Jie *et al.* [35], those patients scoring 5 or higher on the NRS 2002 malnutrition scale received the most benefit from perioperative nutritional support.

**Table 1:** Nutrition Risk Screening (NRS) 2002. Note: This table is reproduced and adapted with permission from [30], Copyright © 2003 Elsevier Ltd.

Nutritional Risk Scoring (NRS)				
***Initial Screening***				
		Yes	No	
Is BMI < 20.5?				
Has the patient lost weight within the last 3 months?				
Has the patient reduced dietary intake in the last week?				
Is the patient severely ill (e.g., in intensive therapy)?				
Yes: If the answer is “Yes” to any question, the final screening is performed.				
No: If the answer is “No” to all questions, the patient is re-screened at weekly intervals. If the patient, e.g., is scheduled for a major operation, a preventative nutritional care plan is considered to avoid the associated risk status.				
***Final Screening***				
	***Impaired Nutritional Status***			***Severity of Disease (≈Increase in Requirements)***
Absent Score 0	Normal Nutritional Status	Absent Score 0		Normal Nutritional Requirements
Mild Score 1	Wt loss >5% in 3 months or Food intake below 50%–75% of normal requirement in preceding week	Mild Score 1		Hip fracture * Chronic patients, in particular with acute complications: Cirrhosis *, COPD *. Chronic hemodialysis, diabetes, oncology
Moderate Score 2	Wt loss >5% in 2 months or BMI 18.5–20.5+ impaired general condition or food intake 25%–60% of normal requirement in preceding week	Moderate Score 2	Major abdominal surgery * Stroke * Severe pneumonia, hematologic malignancy	
Severe Score 3	Wt loss >5% in 1 month (>15% in 3 months) or BMI > 18.5+ impaired general condition or Food intake 0%–25% of normal requirement in preceding week in preceding week.	Severe Score 3	Head injury * Bone marrow transplantation * Intensive care patients (APACHE410)	
Score	+	Score	=Total score:	
Score ≥3: The patient is nutritionally at-risk and a nutritional care plan is initiated.				
Score <3: Weekly rescreening of the patient. If the patient, e.g., is scheduled for a major operation, a preventive nutritional care plan is considered to avoid the associated risk status.				
* Indicates that a trial directly supports the categorization of patients with that diagnosis.				

**Figure 2:**
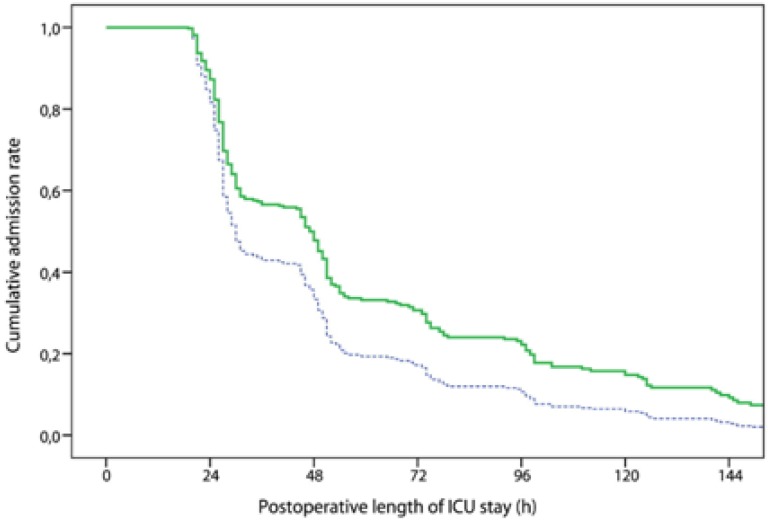
Postoperative admission rates to the intensive care unit among patients undergoing cardiac surgery with low fat-free mass index (green line) and without low fat-free mass index (blue dotted line). This figure is reproduced with permission from [38], Copyright © The American Association for Thoracic Surgery.

Interestingly, malnutrition can occur in obese patients who have low muscle mass. This form of obesity termed sarcopenic obesity may be less recognizable in many cases [36,37]. In many patients fat-free mass index may be a better predictor for mortality than body mass index. Van Venrooij *et al.* [38] found that low fat-free mass index was associated with increased occurrence of adverse outcomes after cardiac surgery [38]. See Figure 2. They advocate fat-free mass index as the leading parameter in classifying and treating malnourished cardiac surgical patients [38].

Options for addressing malnutrition in preoperative patients include dietary counseling, oral supplementation, and artificial feeding. It is estimated that the average survival time for patients at home that do not address their nutritional needs is 19 days [6,39]. Perioperative nutritional intervention, therefore, becomes of great interest and value to both the patient and the surgeon for improving postoperative outcomes.

When designing a nutritional plan for a patient at risk one should ensure that the program is of sufficient duration and intensity to improve known markers of malnutrition [40]. Patient age, sex, height, weight and clinical status, in addition to a detailed medical history and medication list, should be taken into account.

## 3. Parenteral Nutrition

Total parental nutrition (TPN) via central venous catheterization was developed in the late 1960’s by Dr. Stanley Dudrick to provide nutritional support when patients for a variety of reasons were incapable of absorbing nutrients via the gastrointestinal tract [34,41,42]. Initially, its use was hampered by complications such as phlebitis in patients receiving hyperosmotic solutions [41]. Other complications encountered included pneumothorax, subclavian vein thrombosis and septicemia, making the use of TPN limited to patients with extreme malnutrition due to increased risk of administration [41]. However; parenteral formulations and technical advancement have made TPN widely available to most patients.

### 3.1. Benefits

Total parenteral nutrition has been shown to significantly affect postoperative outcomes in the severely malnourished patient group [1,43]. Due to its direct central venous administration, parenteral nutrition can rapidly improve nitrogen balance, which allows for quicker lymphocyte recovery, and improved wound healing [1,6,34,44]. With the addition of vitamins and trace elements, decreases in both infectious and non-infectious complications have been demonstrated [1,45]. Table 2 provides a breakdown of the daily vitamin and trace element requirements for an adult receiving artificial nutrient supplementation. Specifically in 1991, Veterans Affairs completed a study regarding the delivery of preoperative total parenteral nutrition. They demonstrated that preoperative nutritional supplementation with TPN decreased postoperative non-infectious complications from 42.9% to 5.3%. Although found to be beneficial for the severely malnourished, parenteral nutrition for well nourished, or mildly depleted patients, has been shown to have a greater morbidity and should not be used [1].

**Table 2:** The daily vitamin and trace element requirements for an adult receiving artificial nutrition. Note: This table is reproduced with permission from [20], Copyright © 2009 European Society for Clinical Nutrition and Metabolism.

Vitamin/Trace Element	Requirement
**Thiamin (B1)**	6 mg
**Riboflavin (B2)**	3.6 mg
**Niacin (B3)**	40 mg
**Folic Acid **	600 μg
**Panthotenic Acid**	15 mg
**Pyridoxine**	6 mg
**Cyanocobalamin (B12)**	5 μg
**Biotin**	60 μg
**Ascorbic Acid (C)**	200 mg
**Vitamin A**	3300 IU
**Vitamin D**	200 IU
**Vitamin E**	10 IU
**Vitamin K**	150 μg
**Chromium**	10–15 μg
**Copper**	0.3–0.5 mg
**Iron**	1.0–1.2 mg
**Manganese**	0.2–0.3 mg
**Selenium**	20–60 μg
**Zinc**	2.5–5 mg
**Molybdenum**	20 μg
**Iodine**	100 μg
**Fluoride**	1 mg

### 3.2. Risks and Complications of TPN

Although TPN has many benefits, there are considerable risks to its use. Hyperglycemia, along with its metabolic consequences can result in adverse outcomes if allowed to remain uncorrected. Additionally, volume overload can cause respiratory compromise particularly in individuals with marginal cardiopulmonary reserve [45]. Hyperglycemia is also associated with the dysfunction of the immune response. Abnormalities include those affecting granulocyte adhesion, chemotaxis, phagocytosis, respiratory burst function, complement function and intracellular killing [18,45,46,47,48]. It is therefore of no surprise that Compher *et al*. [43]were able to demonstrate that tight glucose control in ICU patients receiving TPN resulted in fewer infectious complications and a decrease in mortality [43]. Overfeeding is another concern with TPN especially in patients at extreme ages or those who are very small or very big. Overfeeding can lead to azotemia, hypertonic dehydration, and metabolic acidosis [49]. Excessive carbohydrate infusion results in hyperglycemia, hypertriglyceridemia, and hepatic steatosis. High lipid infusions can cause hypertriglyceridemia and fat-overload syndrome [49]. Hypercapnia and refeeding syndrome may also result from aggressive feeding [49]. A summary of the complications associated with TPN can be found in Table 3.

**Table 3:** Complications associated with total parenteral nutrition.

**Catheter Insertion Complications**
Arterial puncture
Pneumothorax
Hemothorax
Catheter & wire tip embolization
Air embolism
Thoracic duct injury
Catheter malposition
Cardiac arrhythmias
Mediastinal air/hematoma
Cardiac perforation
Brachial plexus injury
**Catheter Related Complications**
Subclavian vein, internal jogular vein or Superior vena cava thrombosis
Catheter site infection
Septic phlebitis
Catheter associated blood stream infection
**Metabolic Complications**
Hyperglycemia or hypoglycemia
Ketoacidosis
Azotemia & Hyperosmolar state
Electrolyte imbalance
Hypertriglyceridemia
Metabolic acidosis
Hepatic dysfunction
Fluid overload
Coagulopathy

### 3.3. Preoperative TPN Use

If a patient is clinically deemed to be malnourished, then a 7–10 day course of preoperative nutrition is recommended [1,33]. One obvious constraint to this approach is a prolongation in hospital stay if nutrition is given parenterally. As a solution to this, trained nurses can now administer TPN at home and provide close follow up for these patients. Although, preoperative TPN may lower complications postoperatively, it has not been shown to decrease morbidity or mortality [6,50].

### 3.4. Postoperative TPN Use

It is expected that oral food consumption will resume promptly after surgical intervention [3]. Traditionally postoperative nutritional support is, thus, recommended when patients are unable to consume food orally by postoperative day 7–10 if previously well nourished, and postoperative day 5–7 in those previously malnourished prior to surgery [3,20,34]. Routine administration of TPN postoperatively, however, has not been shown to have beneficial effects clinically and may be actually associated with as much as 10% increase in the complication rate [6,50]. Given its risk to benefit profile, parenteral nutrition is therefore not recommended for routine postoperative use.

In summary, when reviewing the literature published to date on the use of perioperative TPN, indications remain unclear as to when and how TPN should be used in the surgical patient. Studies remain inconsistent according to a meta-analysis on perioperative TPN by Heyland *et al.* [50], studies prior to 1988 show a decreased death rate when TPN was used. By contrast, studies published after 1989 do not demonstrate a benefit with the exception of the Veterans Affairs study [50]. As such, it is important to compare the relative advantages of TPN to other forms of nutrition support in the surgical patient.

## 4. Enteral Nutrition

TPN has been the favored route of artificial nutrition until the early nineties; when the benefits of enteral nutrition (EN) became increasingly recognized [42]. Catheter complications and overfeeding with TPN seem to be the two factors that make it less favorable to EN [42].

### 4.1. Benefits

Specific benefits to perioperative EN include a reduction in the incidence of postoperative infections and complications, as well as improved wound healing [1,6,18,51,52]. This would also include fewer life threatening surgical complications, such as anastomotic stenosis or leak, delayed gastric emptying, recurrent nerve palsy, and superficial or deep fascial surgical site infections [8,18]. EN has been shown to be cost effective by reducing the length of hospital stay [8]. These effects are thought to be due to EN capacity to maintain gastrointestinal integrity thus preventing villous atrophy, to attenuate the body’s response to stress and maintain immunocompetency through IgA secretion [1,3,8,53]. EN Contraindications include the presence of intestinal obstruction, malabsorption, multiple fistulas with high output, intestinal ischemia, severe shock with impaired splanchnic perfusion, and fulminant sepsis [20,33,54].

### 4.2. Risks and Complications of EN

Generally complications of EN can be divided into gastrointestinal, mechanical, and metabolic complications. It is important to thoroughly assess patients prior to initiation of tube feeding and to closely monitor them while they are receiving tube feedings in order to identify these potential problems. A summary of the complications associated with EN can be found in Table 4.

**Table 4:** Complications associated with enteral nutrition.

**Mechanical Complications**
Aspiration
Tube malposition
Tube clogging
**Gastrointestinal Complications**
Nausea and vomiting
Diarrhea or constipation
Malabsorption/maldigestion
**Metabolic Complications**
Hyperglycemia or hypoglycemia
Electrolyte imbalance
Early satiety
Dehydration
Refeeding syndrome

### 4.3. Preoperative Use

As previously mentioned, patients found to be clinically malnourished, may require a 7–10 day course of preoperative nutrition [1,33]. If the gut is functioning, the enteral route is preferred over the parenteral route, provided that the patient can tolerate the feeds. Routine preoperative EN supplementation is however unnecessary and of no benefit unless specific nutritional deficiencies are identified.

### 4.4. Postoperative Use

According to Woods *et al.* [55], small bowel function returns approximately 6–8 h after surgery and, despite previous assumptions, a moderate amount of absorptive capacity is present even in the absence of peristalsis [1,55]. Thus, EN in the early postoperative period is not only safe but also beneficial. Although concerns for ileus and anastomotic leak have been raised, there is currently no data to suggest that enteral feeding early postoperatively is responsible for either of these problems [1,6]. In fact some Studies have shown that early enteral nutrition is both effective and well tolerated, although minor complications from this form of nutritional support such as diarrhea and vomiting are seen [1,6]. The decision to institute EN postoperatively revolves around whether the patient has a normally functioning gut. If yes, then it is recommended to make use of the patient’s normal physiology. In a well nourished surgical patient however, a window of about five days is acceptable and EN is not indicated [20,43].

## 5. TPN *vs.* EN

Overall, EN is associated with fewer complications, a decrease in the length of hospital stay, and a favorable cost-benefit analysis, compared to TPN [1,3,56]. The concerns for sepsis and immune dysfunction accompanying the use of TPN are not present EN [34].These points would appear to make EN the superior choice, despite the fact that patients often prefer to be fed intravenously, in order to avoid a nasal feeding tube [5,56]. Additionally, some patients with a working gut may not be able to tolerate oral or enteral feeds due to severe anorexia, dysgeusia, and early satiety and TPN is an acceptable alternative [5].

### Cost Effectiveness

In 1987, cost estimates for the usage of TPN ranged from $75–503/day at a time when patients were required to remain hospitalized [41]. Bozzetti *et al.* [56] in 2001 compared EN and TPN at which time daily costs were in the range of $22 and $53, respectively [56]. Another study completed by Braga *et al.* [3] reported a $65/day saving and an overall saving of $845 when using EN as opposed to TPN [3]. Currently, there is a broad nature of commercial enteral and parenteral nutritional products and services. In general, enteral formulations remain cheaper to administer than those given intravenously. A variety of home nutritional services are now available providing state-of-the-art care for patients requiring aggressive nutrition in any form.

## 6. Combined EN and TPN

The benefits of additional EN to TPN in surgical and critically ill patients are not clear. However, it may be a reasonable approach in patients who can tolerate limited amount of EN due to gastrointestinal dysfunction. A randomized controlled trial by Heidegger *et al.* [57] showed that combining TPN with EN after day 4 of ICU admission in patients for whom EN is insufficient to meet their nutritional goals have reduced nosocomial infections and improved their clinical outcomes [57]. A small retrospective study showed that combined enteralparenteral nutrition in patients with severe acute pancreatitis not only can improve the natural history of pancreatitis but also can reduce the incidence of complication and mortality [58]. In another small retrospective study by Hsu *et al.* [59] surgical intensive care unit patients who could be fed enterally more than 10% of total calories had better clinical outcomes [59]. In an animal study, Omata and colleagues 26 found that EN could reverse TPN-induced impairment of hepatic immunity. They suggested that enteral feeding should be given to induce recovery of hepatic immunity and reduce infectious complications [60]. Although larger scale studies are yet to confirm many of these findings, enteral feeding should be highly considered whenever possible in severely ill patients.

## 7. Immunonutrition

Immunonutrition (IN) is a conceptual framework which enhances enteral nutrition with arginine, omega 3 polyunsaturated fatty acids, glutamine or ribonucleic acid thought to enhance the immune fucntion [44,54,61,62]. A study by Zhang *et al.* [62], demonstrated a reduction in postoperative infection as well as a decrease in length of stay when Immunonutrition was administered postoperatively [62]. Whether IN truly make a difference in postoperative outcomes, remains open to questions. 

Glutamine, in particular, an important and abundant amino acid found both intra- and extracellularly, is essential for nitrogen transport, acid-base homeostasis, and energy delivery in rapidly dividing cells [1]. The preservation of small bowel function and enhanced T lymphocyte responsiveness are seen in the presence of increased glutamine concentrations [33,63,64]. Under extreme stress such as surgery, the demand for glutamine can significantly outweigh the body’s capacity to synthesize this amino acid. Studies are ongoing to determine whether supplementation of glutamine can improve postsurgical outcomes. 

Arginine supplementation is another area under investigation. The hypothesis is that this amino acid, as it is a precursor for nitrous oxide, affects postoperative cardiovascular stability and thus has a role in regulating cardiac and vascular function [10,33]. Like glutamine, arginine also helps the body’s immune response by stimulating T cell function and can even augment the activity of chemotherapeutic agents in cancer patients requiring both surgery and adjuvant therapy [5,33].

Definitive studies proving the benefit of IN are currently available. Bozzetti *et al*. [56] demonstrated that IN was responsible for a two-day decrease in hospital stay, as well as decreasing infection and complication rates [56]. Randomized control trials will be required to demonstrate the superiority of IN over both EN and TPN.

## 8. Conclusion

Since the first randomized trials comparing TPN to the then standard of care which was intravenous normal saline infusion, much progress in nutritional support has been made. In addition, a deeper understanding of the physiologic derangements in surgical nutritionally deficient patients allows the current clinical practitioners to identify patients preoperatively at risk for nutritionally related complications. Improvements in techniques and equipments as well as formulations have made parenteral nutrition safer and effective. The enteral route however, continues to be the optimal approach to aggressive supplemental nutrition in those patients capable of this mode of administration. 

The current recommendation by the ESPN is to employ EN in all patients without contraindications who require nutritional support. The surgical patient with established malnutrition should begin aggressive nutrition at least 7–10 days prior to surgery. Those patients in whom eating is not anticipated beyond the first five days following surgery should receive the benefits of early enteral or parenteral feeding depending on whether the gut can be used. Many patients may benefit from newer enteral formulations, such as those designed to enhance immune function (Immunonutrition), as well as other disease-specific formulations, such as pulmonary insufficiency and renal dysfunction.
